# Does early gonadectomy increase the risk of cranial cruciate ligament disease in female dogs?

**DOI:** 10.18849/ve.v9i2.678

**Published:** 2024-04-25

**Authors:** Daniel Low+

**Keywords:** CANINE, CRUCIATE LIGAMENT, DOGS, EARLY GONADECTOMY, EARLY NEUTERING, RISK

## Abstract

**PICO question:**

In bitches, does gonadectomy before 1 year of age compared to gonadectomy at 1 year of age or older, increase the risk of cranial cruciate ligament disease during their life?

**Clinical bottom line:**

**Category of research:**

Risk.

**Number and type of study designs reviewed:**

Eight studies were reviewed, four of which were retrospective cohort studies, two of which were prospective longitudinal cohort studies, one of which was a retrospective case-control study and one of which was a prospective case-control study.

**Strength of evidence:**

Moderate.

**Outcomes reported:**

The evidence was mixed, but overall was suggestive that gonadectomy before 1 year of age increased the risk of cranial cruciate ligament disease in bitches. Gonadectomy before 1 year of age was found to increase the risk of cranial cruciate ligament disease in seven of eight studies. Most studies assessed a narrow range of breeds and the generalisability of these results to all dogs is thus limited. The one study which assessed a range of 35 breeds noted an increase in risk of cranial cruciate ligament disease in 6 of 35 breeds associated with gonadectomy before 1 year of age. Direct comparison of studies is limited by the varying age stratifications, breed analyses, and study methodologies.

**Conclusion:**

Gonadectomy before 1 year of age in Golden Retriever, German Shepherd, and Rottweiler bitches may increase the risk of cranial cruciate ligament disease. In other breeds, limited evidence is available to draw conclusions.

## How to apply this evidence in practice

The application of evidence into practice should take into account multiple factors, not limited to: individual clinical expertise, patient’s circumstances and owners’ values, country, location or clinic where you work, the individual case in front of you, the availability of therapies and resources.

Knowledge Summaries are a resource to help reinforce or inform decision-making. They do not override the responsibility or judgement of the practitioner to do what is best for the animal in their care.

## Clinical scenario

You are a veterinary surgeon in first opinion practice conducting a health check and vaccination of a female puppy. You take the opportunity to discuss various aspects of routine and preventative healthcare with the clients, including gonadectomy. The client has read online that gonadectomy causes cranial cruciate ligament disease (CCLD) and is seeking your professional opinion on the matter.

## The evidence

Eight studies relevant to the PICO question were reviewed (Ekenstedt et al., 2017; Hart et al., 2014; Hart et al., 2016; Hart et al., 2020; Simpson et al., 2019; Torres de la Riva et al., 2013; Whitehair et al., 1993; and Waters et al., 2023). Overall, the strength of evidence is of moderate quality, due to limitations in study design and methodologies.

## Summary of the evidence

**Ekenstedt et al. (2017):** 

Population	Labrador Retrievers presenting to one of four specialty referral hospitals in North America between 2010 and 2011.
Sample Size	313 Labrador Retrievers, of which 168 were female Labrador Retrievers (83 neutered at or before 1 year of age, 25 neutered after 1 year of age, 60 intact). The results of the male dogs do not relate to the PICO question and therefore will not be commented on further in this Knowledge Summary.
Intervention Details	174 Labrador Retrievers were recruited as cranial cruciate ligament disease (CCLD) cases and 139 Labrador Retrievers were recruited as controls (no CCLD). Data collected included: Gonadectomy status. Age at gonadectomy if relevant. Presence of a mutation in the dynamic 1 gene (DNM1). Fisher’s exact test was used.
Study design	Prospective case-control study.
Outcome studied	Primary outcome studied: association between presence of DNM1 and CCLD.Secondary outcome studied: association between gonadectomy status, time of gonadectomy and risk of CCLD compared to control population.
Main findings (relevant to PICO question)	In the CCLD group, 59/83 (71%) bitches were gonadectomised at or before 1 year of age and 9/25 (36%) bitches were gonadectomised after 1 year of age. In the control group, 24/40 (60%) bitches were gonadectomised at or before 1 year of age and 16/40 (40%) bitches were gonadectomised after 1 year of age. There was a significant difference between groups when comparing bitches gonadectomised at or before 1 year of age, with bitches gonadectomised after 1 year of age, on risk of CCLD (P = 0.0021, odds ratio 4.30 [1.55–12.72]).
Limitations	Evaluating association between gonadectomy, gonadectomy timing, and CCLD was not a primary objective of the study. Narrow study population of dogs presented to tertiary referral hospitals not necessarily representative of the general canine population. Breed-specific study not representative of the general canine population.

**Hart et al. (2014):** 

Population	Labrador Retrievers and Golden Retrievers who presented to the University of California-Davis, Veterinary Medical Teaching Hospital, between 1 January, 2000 and 31 December, 2012. Cases were excluded if records were incomplete. Dogs younger than 1 year of age or older than 9 years of age were excluded.
Sample Size	1015 Golden Retrievers, of which 472 female Golden Retrievers (306 neutered, 166 intact). 1500 Labrador Retrievers, of which 692 female Labrador Retrievers (347 neutered, 345 intact).The results of the male dogs do not relate to the PICO question and therefore will not be commented on further in this Knowledge Summary.
Intervention Details	Data collected included: Gonadectomy status, with intact bitches serving as the control group. Age at gonadectomy if relevant. Diagnosis of cranial cruciate ligament disease (CCLD). Method of diagnosis. Diagnosis of hip dysplasia, elbow dysplasia, lymphoma, haemangiosarcoma, mast cell tumour, mammary cancer. A Cox proportional hazards model was applied, with CCLD tear as the outcome. Timing of gonadectomy within the gonadectomised group was not reported. A case may be included in the intact group if disease occurred before gonadectomy and may then be included in the gonadectomised group if disease occurred after gonadectomy.
Study design	Retrospective cohort study.
Outcome studied	Age at gonadectomy and risk of CCLD, hip dysplasia, elbow dysplasia, lymphoma, haemangiosarcoma, mast cell tumour, and mammary cancer compared to control population of intact dogs of a specific breed.
Main findings (relevant to PICO question)	Golden Retrievers Group A: 11/101 (10.89%) of Golden Retriever bitches neutered gonadectomised before 6 months of age developed CCLD tear. Group B: 4/81 (4.94%) of Golden Retriever bitches neutered gonadectomised between 6 and 11 months of age developed CCLD. Group C: 0/32 (0%) of Golden Retriever bitches neutered gonadectomised at 1 year of age developed CCLD. Group D: 3/89 (3.37%) of Golden Retriever bitches neutered gonadectomised between 2 and 8 years of age developed CCLD tear. One Golden Retriever bitch was excluded for analysis. 0/165 (0%) of intact Golden Retriever bitches developed CCLD tear. Groups A, B, and D had a significantly increased risk of CCLD tear compared to intact controls. A p-value range was reported (< 0.001–0.03) but not specified for each group, and no hazard ratio was reported. Labrador Retrievers Group A: 3/59 (5.08%) of Labrador bitches gonadectomised before 6 months of age developed CCLD. Group B: 5/101 (4.95%) of Labrador bitches gonadectomised between 6 and 11 months of age developed CCLD. Group C: 0/50 (0%) of Labrador bitches gonadectomised at 1 year of age developed CCLD. Group D: 1/128 (0.78%) of Labrador bitches gonadectomised between 2 and 8 years of age developed CCLD. Two Labrador bitches were excluded for analysis. 8/343 (2.33%) of intact Labrador bitches developed CCLD. No significant increase in risk of CCLD in neutered Labrador Retriever bitches, regardless of timing of gonadectomy, compared to intact controls.
Limitations	Gender bias of study population not necessarily representative of the general population of Golden Retrievers or Labrador Retrievers. Timing of gonadectomy was not consistently available and losses prior to data analysis were present. Arbitrary exclusion of cases 9 years or older biases the sample population. Narrow study population of dogs presented to tertiary referral hospital not necessarily representative of the general canine population. Breed-specific study not representative of the general canine population.

**Hart et al. (2016):** 

Population	German Shepherd dogs who presented to the University of California-Davis, Veterinary Medical Teaching Hospital between 1 January, 2000 and 30 June, 2014.Cases were excluded if records were incomplete. Dogs younger than 1 year of age or older than 9 years of age were excluded.
Sample Size	1170 dogs, of which 705 male and 465 female. Of the 465 females, 293 were neutered and 172 were intact. The results of the male dogs do not relate to the PICO question and therefore will not be commented on further in this Knowledge Summary.
Intervention Details	Data was collected from the computerised medical records at the University of California-Davis, Veterinary Medical Teaching Hospital. Data collected included: Gonadectomy status, with intact bitches serving as the control group. Age at gonadectomy if relevant. Diagnosis of cranial cruciate ligament disease (CCLD). A Cox proportional hazards model, or Kaplan-Meier life table analysis if the former was not applicable, was applied, with CCLD tear as the outcome. Timing of gonadectomy within the gonadectomised group was not reported. A case may be included in the intact group if disease occurred before gonadectomy and may then be included in the gonadectomised group if disease occurred after gonadectomy.
Study design	Retrospective cohort study.
Outcome studied	To determine the effect of a single IV injection of paracetamol on the MAC of sevoflurane in response to noxious mechanical stimuli in dogs.
Main findings (relevant to PICO question)	Group A: 2/44 (4.3555%) of bitches gonadectomised before 6 months of age developed CCLD. Group B: 7/84 (8.33%) of bitches gonadectomised between 6 and 11 months of age developed CCLD. Group A + B: 9/128 (7.03%) of bitches gonadectomised before 1 year of age developed CCLD. Group C: 0/36 (0%) of bitches gonadectomised at 1 year of age developed CCLD. Group D: 1/95 (1.05%) of bitches neutered gonadectomised between 2 and 8 years of age developed CCLD. Thirteen bitches were excluded for analysis. 1/159 (0.63%) of intact bitches developed CCLD. (Group A + Group B) had a significantly increased risk of CCLD compared to intact bitches (P = 0.0063, hazard ratio 3.97 [1.48–10.71]). All other comparisons were not significant.
Limitations	Gender bias of study population not necessarily representative of the general population of German Shepherd dogs. Arbitrary creation of a composite group to obtain statistical significance. Arbitrary exclusion of cases 9 years or older biases the sample population. Narrow study population of dogs presented to tertiary referral hospital not necessarily representative of the general canine population. Breed-specific study not representative of the general canine population.

**Hart et al. (2020):** 

Population	Hospital records of dogs of 35 breeds presented to the University of California-Davis, Veterinary Medical Teaching Hospital over a 15 year undefined time period. Cases were excluded if records were incomplete, including those without data on age at time of gonadectomy. Dogs were excluded if the disease of interest occurred at < 12 months of age.
Sample Size	4580 gonadectomised females, 3001 entire females, 3925 gonadectomised males, 4458 entire males.The results of the male dogs do not relate to the PICO question and therefore will not be commented on further in this Knowledge Summary.
Intervention Details	Data collected included: Gonadectomy status, with intact bitches serving as the control group. Age at gonadectomy if relevant. Diagnosis of cranial cruciate ligament disease (CCLD) and age at diagnosis. A Kaplan-Meier survival analysis model was used, followed by a log-rank and generalised Wilcoxon test.
Study design	Retrospective cohort study.
Outcome studied	Age at gonadectomy and risk of CCLD tear compared to control population of intact dogs of the same breed.
Main findings (relevant to PICO question)	With respect to bitches, the following findings are reported: Australian Cattle dog: risk of at least one joint disorder significantly increased (15%) if gonadectomised at < 6 months of age (P < 0.05), but no detectable increase in risk of CCLD when analysed individually against timing of gonadectomy. German Shepherd dog: risk of at least one joint disorder significantly increased (20%, 15%, 5%) if gonadectomised at <6 months (P < 0.01), 6–11 months (P < 0.01), and 1–2 years (P < 0.05) respectively, but no detectable increase in risk of CCLD when analysed individually against timing of gonadectomy. Golden Retriever: risk of at least one joint disorder significantly increased (18%, 11%) if gonadectomised at < 6 months and 6–11 months respectively (P < 0.01 for both comparisons), but no detectable increase in risk of CCLD when analysed individually against timing of gonadectomy. Labrador Retriever: risk of at least one joint disorder significantly increased (11%) if gonadectomised at < 6 months and 6–11 months respectively, but no detectable increase in risk of CCLD when analysed individually against timing of gonadectomy. A composite age group gonadectomised < 1 year was arbitrarily created for statistical analysis, which yielded a significant difference (P < 0.01). Rottweiler: risk of at least one joint disorder significantly increased (43%) if gonadectomised at < 6 months (P < 0.05), but no detectable increase in risk of CCLD when analysed individually against timing of gonadectomy. The authors conclude that gonadectomy was associated with CCLD without supportive statistical significance. Saint Bernard: risk of at least one joint disorder significantly increased (100%) if gonadectomised at < 6 months (P <0.01), but no detectable increase in risk of CCLD when analysed individually against timing of gonadectomy. No significant difference in CCLD risk detected in analyses of remaining 29 breeds.
Limitations	Exact p-values were not reported and sometimes reported as a range only. Exclusion of dogs without a date of gonadectomy introduces heavy bias. Use of ‘joint disorders’ as the composite outcome limits dataset applicability to specific disorders within the composite outcome. Arbitrary creation of a composite age group in the Labrador Retriever analysis in order to obtain statistical significance. Small sample size within the less common breeds.

**Simpson et al. (2019):** 

Population	Female Golden Retrievers in the United States enrolled in the Golden Retriever Lifetime Study (Guy et al., 2015). Cases were excluded if they were overweight or had orthopaedic injuries prior to enrollment.
Sample Size	2764 Golden Retrievers, of which 1378 were female. Of the 2764 dogs of both genders, 1246 were intact, 273 were gonadectomised younger than 6 months of age, 577 were gonadectomised between 6–12 months of age, 658 were gonadectomised after 12 months of age. Gender distribution within each group was not reported.
Intervention Details	Data collected included: Gonadectomy status, with intact bitches serving as the control group. Age at gonadectomy if relevant. Veterinary diagnosis of osteoarthritis. Veterinary diagnosis of cranial cruciate ligament disease (CCLD). Body condition score. A Cox proportional hazards model was applied, with either osteoarthritis or CCLD as the outcome.
Study design	Prospective longitudinal cohort study.
Outcome studied	Age at gonadectomy and risk of osteoarthritis or CCLD compared to control population of intact Golden Retrievers.
Main findings (relevant to PICO question)	Ten bitches were excluded for analysis. 41/2754 diagnoses of CCLD and 22/2754 diagnoses of osteoarthritis. Gonadectomy at less than 6 months of age was significantly associated with increased risk of osteoarthritis or CCLD (P < 0.0001, adjusted hazard ratio 4.06 [2.15–7.67]) Gonadectomy at 6–12 months of age (P = 0.16, adjusted hazard ratio (HR) 1.62 [0.83–3.18]), or gonadectomy after 12 months of age (P = 0.28, adjusted hazard ratio 0.64 [0.28–1.44]) was not associated with an increased risk of osteoarthritis or CCLD, compared to the control population. All gonadectomy groups were at increased risk of obesity (≤ 6 months, HR: 1.81, 95% CI: 1.36–2.40), P < 0.0001; > 6 months to ≤ 12 months, HR: 2.21, 95% CI: 1.77–2.73, P < 0.0001; > 12 months, HR: 1.56, 95% CI: 1.24–1.96, P < 0.0001).
Limitations	Gender distribution within each group was not reported and gonadectomy groups included both male and female collectively. Lack of stratification of CCLD and osteoarthritis into separate groups, analysed collectively as ’orthopaedic injury’. Diagnosis of osteoarthritis extracted from medical records is of limited specificity and includes other affected joints. No specific definition of osteoarthritis or criteria to diagnose was reported. While declared a prospective study, some dogs may have already been exposed to gonadectomy prior to enrollment. The true incidence of CCLD was likely underestimated if only reliant on veterinary surgeon reporting within medical records. Individuals only followed up to 6 years of age. Recruitment bias present within the Golden Retriever Lifetime Study. Breed-specific study not representative of the general canine population.

**Torres de la Riva et al. (2013):** 

Population	Golden Retrievers who presented to the University of California-Davis, Veterinary Medical Teaching Hospital between 1 January, 2000 and 31 December, 2009. Cases were excluded if records were incomplete. Dogs younger than 1 year of age or 9 years and older were excluded.
Sample Size	759 Golden Retrievers, of which 364 were female Golden Retrievers (172 gonadectomised before 1 year of age, 70 gonadectomised after 1 year of age, 122 intact). A case may be included in the intact group if disease occurred before gonadectomy and may then be included in the gonadectomised group if disease occurred after gonadectomy.The results of the male dogs do not relate to the PICO question and therefore will not be commented on further in this Knowledge Summary.
Intervention Details	Data collected included: Gonadectomy status, with intact bitches serving as the control group. Age at gonadectomy if relevant. Diagnosis of cranial cruciate ligament disease (CCLD). Method of diagnosis (referring veterinarian only, referring veterinarian and teaching hospital, teaching hospital only). Incidence rate estimates of CCLD. A Kaplan-Meier survival analysis model was used, followed by a log-rank and generalised Wilcoxon test.
Study design	Retrospective cohort study.
Outcome studied	Age at gonadectomy and risk of CCLD compared to control population of intact dogs of the same breed.
Main findings (relevant to PICO question)	Group A: 13/169 (7.7%) of bitches gonadectomised before 1 year of age developed CCLD. Three bitches were excluded for analysis. Group B: 0/69 (0%) of Golden Retriever bitches gonadectomised after 1 year of age developed CCLD. One bitch was excluded for analysis. Group C: 0/122 (0%) of intact bitches developed CCLD. Group A had a significantly increased risk of CCLD compared to Groups B and C (P = 0.001 and 0.001, no hazard ratio reported).
Limitations	Gender bias of study population not necessarily representative of the general population of Golden Retrievers. Arbitrary exclusion of cases 9 years or older biases the sample population. Narrow study population of dogs presented to tertiary referral hospital not necessarily representative of the general canine population. Breed-specific study not representative of the general canine population.

**Waters et al. (2023):** 

Population	Rottweilers enrolled on the Exceptional Ageing in Rottweilers Study (EARS). Cases were excluded if concurrent stifle disease was present or if age at gonadectomy was unavailable.
Sample Size	123 Rottweilers, 76 female (23 gonadectomised ≤ 24 months of age). Gender and gonadectomy timing distribution was not reported.
Intervention Details	Data collected included: Gonadectomy status. Age at gonadectomy if relevant. Diagnosis of cranial cruciate ligament disease (CCLD). Incidence rates and relative risk calculated via Dog Years At Risk (DYAR) of CCLD. A Kaplan-Meier survival analysis model was used, followed by a log-rank and Cox proportional hazards model.
Study design	Prospective longitudinal cohort study.
Outcome studied	Cranial cruciate ligament survival and relation to age at gonadectomy or gonadectomy status.
Main findings (relevant to PICO question)	Relative risk of CCLD was 802/197 (4.07) in group gonadectomised at ≤ 24 months compared to those gonadectomised at > 24 months or intact. Gonadectomy at ≤ 6 months of age had the highest risk of CCLD in both unadjusted and adjusted hazard analyses (both P < 0.001). Gonadectomy at 6–12 months of age had a moderate risk of CCLD in both unadjusted and adjusted hazard analyses (both P = 0.02 and 0.04 respectively). Gonadectomy at 12–24 months of age had a moderate risk of CCLD in both unadjusted and adjusted hazard analyses (both P = 0.005 and 0.006 respectively). No significant increase in risk of CCLD in all other groups. Incidence of CCLD distributed by age group was not reported.
Limitations	Diagnosis of CCLD by a single examiner only. No supplemental radiography performed so false negative physical examination possible. Study population not reflective of the general Rottweiler population due to longevity. Enrollment bias in study. Exposure to gonadectomy may have occurred prior to enrollment in study.

**Whitehair et al. (1993):** 

Population	Dogs examined at one of 23 veterinary medical teaching hospitals in the United States, with medical records on the Veterinary Medical Data Base (VMDB) between 1 July, 1967 and 31 March, 1987.
Sample Size	10,769 dogs with cranial cruciate ligament disease (CCLD), and 591,548 dogs without CCLD as the control population. Age at time of gonadectomy was only known for 244 dogs. Gender and gonadectomy timing distribution was not reported.
Intervention Details	Medical records from the VMDB were extracted for dogs between 1 and 15 years of age and categorised as cases (CCLD) or controls (no CCLD) and then further sub-categorised by age, bodyweight, sex, and gonadectomy status. Data collected included: Signalment data. Gonadectomy status. Age at gonadectomy if relevant. Chi-squared test was used.
Study design	Retrospective case-control study.
Outcome studied	Prevalence of CCLD for each group and epidemiological risk factors predisposing to CCLD.
Main findings (relevant to PICO question)	From 244 dogs, there was no difference in prevalence of CCLD in dogs who had been spayed at a young age and dogs whose age group at the time of gonadectomy was the same as their age group at the time of diagnosis (i.e. gonadectomised close to the time of diagnosis).
Limitations	No p-value or other descriptive statistics reported, other than that 244 dogs were available for data analysis. Non-definition of ‘early gonadectomy’. Poorly defined category of ‘late gonadectomy’ – highly variable definition used in this study.

## Appraisal, application and reflection

Eight studies were identified which were directly relevant to the PICO question (Ekenstedt et al., 2017; Hart et al., 2014; Hart et al., 2016; Hart et al., 2020; Simpson et al., 2019; Torres de la Riva et al., 2013; Whitehair et al., 1993; and Waters et al., 2023). The studies were cohort or case-control studies, conducted retrospectively or prospectively, and are only able to provide moderate-quality evidence, at best.

Breed differences and differences in methodologies limits the validity of comparison between studies. Breed-specific studies were evaluated head-to-head, where appropriate, and methodological differences were evaluated. Different measures of outcome were employed between studies. For example, Simpson et al. (2019) evaluated cranial cruciate ligament disease (CCLD) together with osteoarthritis, and Hart et al. (2020) evaluated joint disorders as a composite outcome, in addition to evaluating the primary outcomes of CCLD, hip dysplasia, or elbow dysplasia.

Golden Retriever bitches were evaluated in Simpson et al. (2019), Hart et al. (2014), Torres de la Riva et al. (2013), and Hart et al. (2020). Simpson et al. (2019) reported that in Golden Retrievers, gonadectomy at less than 6 months of age was significantly associated with an increased risk of CCLD or osteoarthritis. Hart et al. (2014) reported that in Golden Retriever bitches, gonadectomy was significantly associated with CCLD, if gonadectomised at less than 1 year of age, or at more than 2 years of age. From Hart et al. (2014), gonadectomy at 1 year of age (12–24 months) was not associated with CCLD. In Hart et al. (2014), it is also notable that 11/101 (10.89%) of Golden Retrievers gonadectomised before 6 months of age developed CCLD, compared to those gonadectomised between 6–11 months (4/81 [4.94%]) and 2–8 years (3/89 [3.37%]). Torres de la Riva et al. (2013) reported an increased risk of CCLD when dogs were gonadectomised before 1 year of age. Finally, Hart et al. (2020) reported that the Golden Retriever was at increased risk of at least one joint disorder when gonadectomised at less than 12 months of age.

For Labrador Retrievers bitches, Hart et al. (2014) did not find a statistically significant difference in risk of CCLD in gonadectomised Labradors compared to intact Labradors, although a trend was noted with gonadectomy under 12 months of age. Ekenstedt et al. (2017) did however find an increased risk of CCLD in their sample of Labrador Retrievers associated with gonadectomy before 12 months of age.    

For Rottweilers, Waters et al. (2023) reported an increased risk of CCLD in all gonadectomy groups when gonadectomised before 24 months of age, with increasing risk the earlier gonadectomy was performed.

The only study to evaluate a large number of various breeds was Hart et al, 2020. However, this study was limited by small numbers of dogs in certain breed groups, especially those of the less common breeds.

Studies differed in cohort definitions, with Hart et al. (2016) and Hart et al. (2014) evaluating early gonadectomy with < 6 month and 6–12 month groups, whereas Torres de la Riva et al. (2013) and Ekenstedt et al. (2017) collectively evaluated these groups together as a < 1 year group. Simpson et al. (2019) did not stratify between sex therefore further comparison was not possible. Hart et al. (2020) defined gonadectomy groups of < 6 months, 6–12 months, 1–2 years, and 2–8 years. However, composite groups were created for statistical analysis, where convenient. Overall, different definitions between studies limits direct comparison.

In general, the majority of studies (Ekenstedt et al., 2017; Hart et al., 2014; Hart et al., 2016; Torres de la Riva et al., 2013; Hart et al., 2020; and Waters et al, 2023) found that spaying < 1 year of age was associated with an increased risk of CCLD, however Hart et al. (2014) also reported that spaying between 2–8 years of age was associated with an increased risk. Whitehair et al. (1993) did not find that early spaying increased the risk of cranial cruciate ligament disease however several methodological limitations were present in this study. Where multiple breeds were analysed in Hart et al. (2020), most breeds were not found to have an increased risk of CCLD with gonadectomy.

Methodological limitations limited the strength of evidence provided by all studies. Simpson et al. (2019) utilised inclusion criteria which would likely have simultaneously under- and over-estimated the true incidence of disease, which was a significant confounding factor. One of two of the longitudinal studies (Simpson et al., 2019) was limited in its length of follow-up and none of the study participants were followed for life, for logistical or study design reasons. Of the one study with lifetime follow-up (Waters et al., 2023), the exceptional longevity of the study population cannot be said to be representative of the general population of Rottweilers. Statistical analysis was also confounded by the use of composite outcomes and composite groups, which limits the direct applicability of the studies to the PICO question. Most of the studies, with the exception of Hart et al. (2020) evaluated one or two breeds of dog, which limits the ability to generalise their findings to the canine population at large. In the studies where gonadectomy status was collected retrospectively (Hart et al., 2014; Simpson et al., 2019; Torres de la Riva et al., 2013; Hart et al., 2020; and Waters et al., 2023), these data may have been subject to incorrect reporting or inaccuracies in recall. Next, despite the relatively large sample sizes, the incidence of cranial cruciate ligament disease was low. Therefore, statistical analysis was performed on small numbers of cases, some of which were in the single digits, and limits the reliability of results. Finally, most of the study participants were drawn from a narrow population of dogs attending veterinary referral hospitals in all but Simpson et al. (2019) and Waters et al. (2023).

Overall, the evidence is mixed and of moderate quality. There is evidence to support the assertion that gonadectomy in bitches before 1 year of age is associated with an increased risk of CCLD in three breeds – the Golden Retriever, German Shepherd, and Rottweiler. There was also contradictory evidence to suggest that gonadectomy in bitches at 6–12 months of age was not associated with CCLD and that gonadectomy in bitches, regardless of timing, was associated with CCLD. There was a trend towards an increased risk of CCLD associated with gonadectomy in bitches, in the narrow range of breeds represented throughout most studies, but particularly in the Golden Retriever. Overall, no firm conclusions can be drawn. Further studies are required, particularly in the subgroup of dogs gonadectomised < 1 year of age.

## Methodology

**Search strategy:** 

Databases searched and dates covered:	CAB Abstracts on OVID Platform covering from 1973 to 2023 Week 45PubMed via the NCBI website covering from 1910 to November 2023
Search strategy:	CAB Abstracts: (dog or dogs or canine or canines or canis or bitch or bitches or puppy or puppies or pups).mp (spay* or spey* or neuter* or ovariohysterectom* or ovario-hysterectom* or ovariectom* or gonadectom* or desex* or de-sex*).mp ((cranial or anterior) and (cruciate or stifle injury)).mp 1 and 2 and 3 Pubmed: (cranial or anterior) and (cruciate or stifle injury) (spay* or spey* or neuter* or ovariohysterectom* or ovario-hysterectom* or ovariectom* or gonadectom* or desex* or de-sex*) (dog OR dogs OR canine OR canines OR canis OR bitch OR bitches OR puppy OR puppies OR pup OR pups) 1 AND 2 AND 3
Dates searches performed:	12 Nov 2023

**Exclusion / inclusion criteria:** 

Exclusion:	Opinion pieces, articles on neuter status and risk of cranial cruciate ligament rupture but not on timing of neutering, articles on timing of neutering but not on cranial cruciate ligament rupture, and articles that were not relevant to the PICO question.
Inclusion:	Articles that were relevant to the PICO question.

**Search outcome:** 

Database	Number of results	Excluded – Opinion pieces	Excluded – Not on timing of neutering	Excluded – Not on risk of cruciate disease	Excluded – Not relevant to the PICO question	Excluded – Not accessible	Total relevant papers
CAB Abstracts	65	9	9	3	38	0	6
PubMed	48	2	10	0	28	0	8
Total relevant papers when duplicates removed							8

## ORCiD

Daniel Low: https://orcid.org/0000-0003-0215-4997

## Conflict of interest

The author declares no conflicts of interest.
